# Biochemical and cytological analyses of pleural effusion in patients with lung anthracosis and antracofibrosis

**DOI:** 10.1016/j.amsu.2021.102686

**Published:** 2021-08-13

**Authors:** Ahmad Shafahi, Mitra Samareh Fekri, Seyed Mahdi Hashemi Bajgani, Rostam Yazdani, Mohsen Shafiepour, Mohammad Hassan Touhidi, Hamid Sharifi, Fatemeh Tayari, Ali Sharifpour

**Affiliations:** a - Department of Internal Medicine, Kerman University of Medical Sciences, Kerman, Iran; b - Cardiovascular Research Centre, Institute of Basic and Clinical Physiology Sciences, Kerman of Medical Sciences, Kerman, Iran; c Kerman County Health Center, Diseases Prevention and Control Unit, Kerman University of Medical Sciences, Kerman, Iran; d HIV/STI Surveillance Research Center, Kerman University of Medical Sciences, Kerman, Iran; e - Kerman University of Medical Sciences, Kerman, Iran; f - Pulmonary and Critical Care Division, Imam Khomeini Hospital, Iranian National Registry Center for Lophomoniasis (INRCL), Mazandaran University of Medical Sciences, Sari, Iran

**Keywords:** Anthracosis, Antracofibrosis, Pleural effusion, Lung

## Abstract

**Background:**

Anthracosis and anthracofibrosis are attributed to the deposition of carbon particles along with fibrosis, adhesion, narrowing, and collapse. There has been no study on the characteristics of the pleural fluid in anthracosis. The present study analyzed the biochemical characteristics of pleural effusion in patients with pulmonary anthracosis.

**Patients and methods:**

The study is a cross-sectional study which included patients who were referred to the Afzalipour Hospital in Kerman, eastern Iran. Between April 2018 and October 2019, patients who had undergone bronchoscopy and were diagnosed with anthracosis and pleural effusion were selected through the census method. The characteristics of the pleural fluid were analyzed for protein, albumin, LDH, PH, Triglyceride, cholesterol, glucose, and cytology. Concomitant blood samples were examined for LDH, albumin, total protein, and glucose. After it was specified whether the pleural effusion was transudative or exudative, patients with lymphocyte-dominant exudative pleural fluid became candidates for thoracoscopy.

**Result:**

106 patients (6.21 %) of 1705 patients had anthracosis and anthracofibrosis; 37 of these patients (34.9 %) had coexisting pleural effusion. 31 patients gave written informed consent for thoracentesis. The mean age of the patients was 76.48 ± 8.81. In addition, 67.74 % of the patients were female. Pleural effusion was transudative in 29 (93.54 %). Except for one case, all patients had diffuse anthracofibrosis and 67.74 % of the patients had a history of baking bread.

**Conclusion:**

According to the findings of this study, most cases had transudative lymphocyte-dominant pleural fluid with mostly diffuse anthracofibrosis. In addition, this condition is more prevalent in women, with a prevalence of approximately twice that of men.

## Highlights

• Biochemical and cytological analyses of pleural effusion in patients with lung anthracosis and antracofibrosis.
• The observation of the transudative fluid with lymphocytosis in pleural effusion of patients with anthracosis.
• In this study, the incidence of anthracnose or anthracofibrosis with pleural effusion was more common in women.

## 1 Introduction

Simple anthracosis is attributed to the deposition of carbon particles in the bronchi and is presented as black pigmentation on bronchoscopy. On the other hand, anthracofibrosis is defined as the deposition of carbon particles along with fibrosis, adhesion, narrowing, and collapse, which can be local or diffuse [1,2]. No specific prevalence rate has been identified for this disease, and different studies have reported varying percentages. For instance, two studies conducted in South Korea and Iran reported 3 % and 21.8 % prevalence, respectively [3,4]. Moreover, in a review study conducted by Mirsadraee, the prevalence of anthracosis and anthracofibrosis was reported to be 4.3–21 % and 0.1–22.5 %, respectively [5]. Pulmonary anthracosis is a type of pneumoconiosis (lung disease caused by mineral particles) caused by the inhalation of carbon particles and leads to the formation of black plaques in the lungs, parenchyma, bronchi, and respiratory bronchioles. This condition is mostly observed in residents of industrial areas, coal mine workers, and people who work with graphite compounds, carbon black, and electrodes and has symptoms similar to those of chronic obstructive pulmonary disease [5,6]. This disease is prevalent in developing countries, but it is rare in industrialized countries, and the majority of people with this disease are immigrants from third world countries [7]. The epidemiology of developing countries shows that exposure to wood smoke can play an etiologic role in a wide range of adult medical diseases [5,8]. Some studies claim that tuberculosis can be a risk factor for developing anthracosis, and in some studies, the comorbidity rate was reported to be 37 % [9]. Another study reported a 25 % comorbidity rate [10]. In two studies in Iran by Samareh Fekri et al. the prevalence of tuberculosis in cases of bronchial anthracosis was 9.4 % and 6.9 % [3,11]. Different coexistence rates of pleural effusion in patients with anthracosis and anthracofibrosis have been reported. However, no study has been conducted on pleural effusion and its characteristics. For instance, in a study conducted by Shafahie et al. in Kerman on 65 patients with anthracofibrosis, the coexistence of pleural effusion was approximately 33 % (21 patients), and in another study in Korea on 14 patients with anthracofibrosis, this percentage was approximately 14 (2 patients) [5,6,11]. Anthracosis and anthracofibrosis make up an average of 10–12 % of bronchoscopy reports in Iran [5]. Moreover, the coexistence of pleural effusion is observed in up to one-third of cases in some reports, but there has been no report regarding the biochemical analysis of pleural fluid in these patients. Therefore, this study aimed to analyze the pleural fluid in patients with anthracosis and anthracofibrosis.

## 2 Patients and Methods

The sampling was done through the census method for 18 months between April 2018 and October 2019. All the patients who had undergone bronchoscopy at Afzalipour Hospital in Kerman, eastern Iran, and had anthracosis or anthracofibrosis with coexisting pleural effusion were included in the study. After informed consent was obtained, the patient's pleural effusion was tapped (Thoracentesis) after examination using medical ultrasound. The tapping area was first prepped and then a 10-cc syringe was inserted into the pleural space, and the pleural fluid was removed to be analyzed in terms of total protein, albumin, LDH, PH, TG, cholesterol, glucose, and cytology. Moreover, concomitant blood samples were also examined in terms of LDH, albumin, total protein, and glucose.

If, according to Light's criteria, at least one of these criteria was met, the pleural fluid was considered exudative: The ratio of pleural fluid protein to serum protein was more than 0.5. The ratio of pleural fluid LDH to serum LDH was more than 0.6. Pleural fluid LDH was two thirds more than the highest normal amount of LDH in the serum and if none of the aforementioned criteria were met, the pleural fluid was considered transudative.

After it was specified whether the pleural effusion was transudative or exudative, patients with lymphocyte-dominant exudative pleural fluid underwent thoracoscopy. In addition, those with transudative pleural effusion were examined to check for coexisting diseases such as heart failure, cirrhosis, nephrotic syndrome, hypothyroidism, hypoproteinemia, and pulmonary embolism, which can cause transudative pleural effusion, and patients who had any of these diseases were excluded from the study. Regarding the evaluation of heart failure in patients with transudative pleural effusion, echocardiography was performed on all patients and every patient with impaired ejection fraction or diastolic dysfunction was excluded from the study. The data was analyzed using SPSS V.22 software and, in all cases, the value of P was less than 0.05 according to the statistical judgment criteria. Then the results were presented using descriptive statistics such as mean, standard deviation, percentage, table and graph. This cross-sectional study was approved by the Kerman University of Medical Science Ethics Committee (IR.KMU.REC.1397.550) and was carried out in accordance with the Helsinki Declaration principles. The work has been reported in line with the STROCSS criteria [12]. This study is registered with the Research Registry, and the UIN is research registry 6916 (https://www.researchregistry.com/register-now?__cf_chl_jschl_tk__=pmd_45c1685c37c7bb3418ea874faa8765e6875c1721-1627701987-0-gqNtZGzNAqKjcnBszQqO#home/registrationdetails/60d1bc85d4aae2002227822d/):

## 3 Results

In this study, 106 patients (6.2 %) of a total of 1705 patients who underwent bronchoscopy during the 18 months had anthracosis and anthracofibrosis; 37 of these patients with anthracosis and anthracofibrosis (34.9 %) had coexisting pleural effusion and 31 of these patients agreed with sampling and pleural fluid analysis and formed the study population. The mean age of the participants was 76.48 ± 8.81 (age range of 61–92). 20 of the participants were housewives (64.5 %), and 21 patients (67.7 %) had a history of bread baking. Furthermore, 3 participants (9.7 %) had a history of tobacco (cigarette) use, and 8 of them (25.8 %) had a history of opium use. According to the cytology results, BAL samples of all patients were negative for BK. Moreover, more than three quarters of the patients had diffuse anthracosis and anthracofibrosis. The analysis showed that in patients with anthracosis and anthracofibrosis, in addition to pleural effusion, pleural fluid was unilateral and bilateral in 17 (54.8 %) and 14 (41.9 %) of patients, respectively. Moreover, the findings of the study showed that 29 patients (93.5 %) had transudative pleural effusion, and only 2 patients (6.45 %) had the exudative type (Table 1).

**Table 1:** Demographic variables of the participants.

Variables	frequency percentage) Frequency)	
Gender	Male	10 (32.2)
	Female	21 (67.7)
Occupation	Housewife	20 (64.5)
	Farmer	6 (19.3)
	Carpet weaver	3 (9.7)
	Retired	2 (6.45)
Bread baking history	Yes	21 (67.7)
	No	10 (32.3)
Tobacco use history	Yes	3 (9.7)
	No	38 (90.3)
Opium use history	Yes	8 (25.8)
	No	23 (74.2)

Pleural effusion cholesterol and triglycerides were 29.77 mg/dl (4–161 mg/dl) and 17.19 mg/dl, respectively, in patients with pulmonary anthracosis and anthracofibrosis. Moreover, the mean of pleural effusion glucose and PH in patients with pulmonary anthracosis and anthracofibrosis were 172.35 mg/dl and 7.58 mg/dl, respectively. In addition, the mean of LDH, protein, and albumin were 223.41, 2.13, and 1.25 mg/dl, respectively (Table 2).

**Table 2:** Frequency of patients with regard to positive BAL in terms of BK, diffuse or local, unilateral or bilateral, and transudative or exudative pleural fluid.

Variables	frequency percentage) Frequency)	
BAL interms of BK	Positive	0 (0)
	Negative	31 (100)
Anthracosis and anthracofibrosis	Local	1 (3.2)
	Diffuse	30 (96.8)
Pleural fluid	Unilateral	17 (54.8)
	Bilateral	14 (41.9)
Pleural effusion	Transudative	29 (93.5)
	Exudative	2 (6.4)

The results showed that the mean of serum glucose, LDH, protein, and albumin were 162.35 ± 84.239, 409.25, 5.87, and 3.40 g/dl, respectively. Analysis of pleural fluid in terms of white blood cell count showed that the lymph type was more frequent than the PMN type (see Table 3, Table 4). Thoracoscopy sampling revealed the coexistence of chronic pleuritis in two patients with lymphocyte-dominant exudative pleural effusion (Table 5).

**Table 3:** Mean of pleural fluid variables in participant.

Variable	Mean	Standard deviation	Minimum	Maximum
Cholestrole	29.77	28.007	4	161
Triglycride	17.19	14.853	4	87
Glucose	172.35	77.480	83	391
PH	7.58	0.179	7.29	8
LDH	223.41	233.855	20	1301
Protein	2.13	0.991	0.7	4.4
Albumin	1.25	0.586	0.4	2.7

**Table 4:** Mean of blood glucose, LDH, protein, and albumin of the participants.

Variable	Mean	Standard deviation	Minimum	Maximum
Glucose	162.35	84.239	78	421
LDH	409.25	222.249	96	1310
Protein	5.87	0.921	3.5	7.5
Albumin	3.40	0.481	2.2	4.2

**Table 5:** Frequency of white cell types in pleural fluid of the patients.

White cell type	Mean
PMN	32.5
Lymph	60.6
Mesothelial	18.8

## 4 Discussion

In this study, 31 patients with anthracosis or anthracofibrosis with coexisting pleural effusion were evaluated. The mean age of the patients was 76.48 ± 8.81. Therefore, it can be assumed that the coexistence of pleural effusion with anthracosis and anthracofibrosis is a complication observed in older people. This could be due to the fact that this disease takes time to change and create symptoms in the lungs. This finding was in accordance with the mean age of patients with anthracosis and anthracofibrosis in studies conducted by Shafahie and Hosseini [Unpublished data], Mirsadraee et al. [5], Aka Akturk et al. [13], and Samareh Fekri et al. [3]. In terms of gender, there were 21 female patients (67.74 %) and 10 male patients (32.26 %). As is evident, the ratio of coexistence of pleural effusion with anthracosis and anthracofibrosis in women to that of men is approximately two. This finding was in accordance with the gender frequency of anthracosis and anthracofibrosis patients [3,5,13]. In terms of occupation, 20 patients (64.52 %) were housewives, and 21 patients (67.74 %) had a history of bread baking. Moreover, all housewives have a history of bread baking, which shows the relationship between bread baking and anthracosis. Accordingly, bread baking can even be considered an anthracosis risk factor. It seems that exposure to fossil fuels and baking and cooking can be risk factors for this disease [3,5,11]. The similarity between prevalence rates in terms of gender, occupation, and age in anthracosis and anthracofibrosis with or without pleural effusion can support the idea that anthracofibrosis is a disease with its own specific coexisting pleural effusion. Three (9.7 %) of the participants were smokers, and 8 (25.8 %) were opium addicts. This statistic demonstrates the absence of a strong link between tobacco use and disease. The bronco alveolar lavage test (smear and culture) revealed that all patients were negative for BK, while 6 (19.35 %) were positive in terms of tuberculosis history. In the study by Mortazavi-Moghaddam et al. [14], among the 89 patients with anthracosis evaluated for the existence of tuberculosis using acid-fast smear, culture, and biopsy, 43 patients were positive for tuberculosis. This finding indicates a strong relationship between tuberculosis and anthracosis. Moreover, in the study by Aka Akturk et al. in which 107 patients were evaluated, 12 patients tested positive for tuberculosis. In two studies in Iran by Samarefekri et al. the prevalence of tuberculosis in cases of bronchial anthracosis was 9.4 % and 6.9 % [3,11]. Therefore, the findings of Mortazavi-Moghaddam et al. [14], Aka Akturk et al. [13], and Samareh Fekri et al. [3,11] are inconsistent with the findings of the present study. However, in the study conducted by Mirsadraee et al. [5], no relationship was found between tuberculosis and anthracosis, which was in accordance with the present study. The reason for this difference may be related to the differences between diagnostic methods and their accuracy. Therefore, it is recommended that more accurate methods, such as PCR, culture, and biopsy, be employed.

Bronchoscopic analysis of different anthracosis types indicated that only one patient (3.2 %) had local anthracosis. On the other hand, 30 patients (96.8 %) had diffuse anthracosis and anthracofibrosis. As a result, the majority of patients with anthracosis and anthracofibrosis had diffuse pulmonary involvement, and the changes caused by fibrosis, adhesion, and airway inflammation most likely resulted in volume reduction and pleural effusion. Regarding the one patient with local anthracofibrosis and pleural effusion, it can be stated that the disease, regardless of the extent of involvement, can cause changes and pleural effusion. Moreover, anthracofibrosis changes can occur in areas out of the bronchoscopes reach, such as smaller tracts. The second presumption has been more acceptable since almost all patients have transudative pleural effusion, and the hydrostatic pressure difference leads to pleural effusion.

The prevalence of pleural effusion with this disease was 34.9 % in the present study. 17 (54.8 %) and 14 (41.9 %) patients had unilateral and bilateral pleural effusions. Shafahi and Hosseini reported a similar prevalence (32.8 %) [Unpublished data], and another study on 14 anthracofibrosis patients in South Korea, reported a coexistence rate of 14% (2 patients) [6]. The coexistence of pleural effusion in more than one-third of patients indicates the significance of the disease in causing pleural effusion, specifically of the transudative type. The analysis of concomitant plasma LDH, protein, albumin and glucose revealed that pleural fluid was transudative and exudative in 29 (93.5 %) and 2 (6.45 %) cases, respectively. Considering the fact that anthracofibrosis can lead to adhesion and collapse in the lung, it is probable that collapse, volume reduction, and an increase in negative pressure in the chest may lead to the accumulation of pleural fluid in the pleural space. Furthermore, if subsequent studies measure the pleural fluid pressure and prove this hypothesis, it can be concluded that removing pleural fluid does not cure the patient, and fluid will accumulate again. Based on these study results, glucose levels are significantly higher in pleural effusion caused by anthracosis, and the pleural fluid is mostly alkaline. The low levels of pleural fluid cholesterol and triglyceride show that anthracosis and anthracofibrosis have no relationship with chylothorax. Moreover, the most pleural effusion cases were lymphocyte-dominant. Cytologic analysis of pleural fluid revealed no malignant cells. The aforementioned finding was in accordance with the findings of Mirsadraee et al. [5]. However, Aka Akturk et al. [13] showed that there is a relationship between anthracosis and lung cancer.

The results of the present study suggest the following measures. First, further studies should be performed on the association of malignancy with pleural effusion due to anthracofibrosis. Failed. Third, evaluation and measurement of pleural fluid pressure, considering the association of tuberculosis with anthracosis, but in this study, BAL smear was not positive in any of the patients. Also, one of the limitations of this study was the dissatisfaction of some patients with the tapping of the pleural fluid and also the lack of a device to measure the pressure of the pleural fluid.

## 5 Conclusion

Based on the findings of the present study, most cases of coexisting pleural fluid in patients with anthracosis and anthracofibrosis were transudative and lymphocyte-dominant and coexisted with diffuse anthracofibrosis. This condition is more prevalent in women, with a prevalence twice that of male patients. Moreover, we recommend conducting similar studies to confirm or reject the findings, conducting more studies on the relationship between malignancy and pleural effusion caused by anthracofibrosis and performing culture or PCR to check for the existence of tuberculosis, since the coexistence of tuberculosis with anthracosis has been previously reported. However, the BAL smear was not positive in any patient and assessed and measured pleural fluid pressure.

## Ethical approval

This cross-sectional study was approved by the Ethics Committee of Kerman University of Medical Sciences (No: IR.KMU.REC.1397.550).

## Sources of funding

The study was funded by the 10.13039/501100004621Kerman University of Medical Sciences. The funder has no role in the design of the study and collection, analysis, and interpretation of data and in writing the manuscript.

## Author contribution

AS and MSF designed the study, wrote the manuscript, and analyzed and interpreted the data. SMHB, RY, MS, MHT, HS, FT and AS, collected the data and provided critical comments. MSF involved in interpretation and editing the manuscript. All authors read and approved the final version of the manuscript.

## Registration of research studies

*6916*.

## Guarantor

Ahmad Shafahi.

## Consent

Not applicable.

## Declaration of competing interest

The authors have no conflicts of interest to declare.

## Availability of data and materials

The authors are responsible for data. Access to all relevant raw data will be free to any scientist.
